# Combining identity by descent and association in genetic case-control studies

**DOI:** 10.1186/1471-2156-9-42

**Published:** 2008-07-05

**Authors:** Qingrun Zhang, Shuang Wang, Jurg Ott

**Affiliations:** 1 Beijing Institute of Genomics, Chinese Academy of Sciences, No. 7 Bei Tu Cheng West Road, Beijing 100029, PR China; 2 Department of Biostatistics, Mailman School of Public Health, 722 West 168th Street, Columbia University, New York, NY 10032, USA; 3 Rockefeller University, Laboratory of Statistical Genetics, 1230 York Avenue, New York, NY 10065, USA

## Abstract

**Background:**

In human case-control association studies, one of the chi-square tests typically carried out is based on a 2 × 3 table of genotypes (homogeneity of three genotype frequencies in case and control individuals). We formulate the two degrees of freedom associated with a given genotype distribution in terms of two biologically relevant parameters, (1) the probability *F *that an individual's two alleles are identical by descent (IBD) and (2) the frequency *p *of one of the alleles.

**Results:**

Imposing the restriction, *F *≥ 0, makes some of the genotype frequencies invalid thereby reducing noise. We propose a new statistical association test, the FP test, by focusing on allele frequency differences between case and control individuals while allowing for suitable IBD probabilities. Power calculations show that (1) the practice of generally carrying out two association tests (allele and genotype test) has an increased type I error and (2) our test is more powerful than conventional genotype and allele tests under recessive trait inheritance, and at least as powerful as these conventional tests under dominant inheritance.

**Conclusion:**

For dominant and recessive modes of inheritance, any apparent power gain by an allele test when carried out in conjunction with a genotype test tends to be purchased entirely by an increased rate of false positive results due to omission of a multiple testing correction. As an alternative to these two standard association tests, our FP test represents a convenient and more powerful alternative.

## Background

In their well-known paper on homozygosity mapping published 20 years ago, Lander and Botstein [1] showed that fewer than a dozen unrelated inbred children should suffice to map a recessive trait given a dense map of genetic markers. They recommended that one should search for extended regions of homozygosity shared by a set of inbred individuals. The power of this approach was demonstrated by the mapping of a gene for a rare recessive trait in a genome-wide investigation of only three distantly related patients [2]: On chromosome 18, five of the six disease chromosomes shared a region of 19 cM in length.

Even individuals seemingly collected at random from the population tend to exhibit extended regions of allele sharing [3], which may be interpreted as the consequence of the mating of (distantly) related individuals. Such homozygosity represents autozygosity, that is, the sharing of two alleles that are copies of a single ancestral allele [3].

In recent years, researchers have shown renewed interest in extended segments of homozygosity and have generally done so by focusing on segments of specific lengths [4-7]. Our purpose here is to focus on individual SNPs rather than on genomic segments of arbitrary lengths, and to allow for biologically reasonable values of IBD while testing for allele frequency differences. The reason for this is not so much biological plausibility but rather statistical power: By disregarding parameter values that are unlikely to be of importance, we reduce the effect of statistical noise and thereby gain accuracy and power. It will be seen below that our approach is somewhat analogous to the "possible triangle" method [8] in affected sib-pair linkage analysis and is expected to lead to similar increases in power.

## Results

### Statistical model for SNP genotype frequencies

Consider a SNP marker with two alleles, *A *and *B*, with *p *= P(*A*). Let *F *denote the probability that the two alleles in an individual are identical by descent (IBD) or autozygous (implies homozygous) or, equivalently, *F *= proportion of individuals who are IBD at that marker, where *F *is the inbreeding coefficient. Given autozygosity (*F *= 1), the frequencies of genotypes *AA*, *AB*, and *BB *are given by *p*, 0, and 1 - *p*, respectively, and given allozygosity (*F *= 0) we assume the corresponding frequencies to be *p*^2^, 2*p*(1 - *p*), and (1 - *p*)^2 ^(Hardy-Weinberg equilibrium, HWE). Then the genotype probabilities may be formulated as given in Table 1.

**Table 1:** Genotype parametrization

*Genotype*	*Frequency*
*AA*	*q*_1 _= *Fp *+ (1 - *F*)*p*^2^
*AB*	*q*_2 _= 2*p*(1 - *p*)(1 - *F*)
*BB*	*q*_3 _= *F*(1 - *p*) + (1 - *F*)(1 - *p*)^2^
*Sum*	1

Parametrization of genotype frequencies (*F *= probability of autozygosity, *p *= frequency of *A *allele).

As there are equal numbers (i.e., 2) of free (independent) genotype classes and parameters, finding maximum likelihood estimates (MLEs) amounts to simply equating genotype frequencies with their probabilities of occurrence (Table 1). Solving the expressions for *q*_1 _and *q*_2 _in Table 1 for *F *and *p *leads to the following solutions:

(1) $$\begin{array}{c} p=q_{1}+q_{2}/2\\ \begin{array}{cc} F=1-q_{2}/[2p(1-p)] & \text{if }p(1-p)>0\\ F=1 & \text{if }p(1-p)=0 \end{array}\} \end{array}\}$$

The MLEs of *q*_1_, *q*_2_, and *q*_3 _are simply the proportions of individuals with given genotypes. Because of the invariance property of MLEs, the functions *p *and *F *of *q*_1 _and *q*_2 _(equation 1) are also MLEs. The inbreeding coefficient *F *may be viewed as an indicator of how far the genotype frequencies deviate from HWE. If *q*_2 _is smaller than expected under HWE then *F *> 0.

The inbreeding coefficient *F *in human populations is known to be small and positive. In North America, it is generally much smaller than 0.01 [9] but in isolated populations may reach value of around 0.10 [10]. However, in samples of individuals affected with a heritable trait, the inbreeding coefficient may be even higher. For example, for the SNP most strongly associated with age-related macular degeneration (AMD) [11], rs380390, application of equations (1) leads to an estimated inbreeding coefficient of 0.13 in case individuals. Such increases occur because of enrichment of a disease genotype due to ascertainment of case individuals, and/or they may be a consequence of the fact that individuals affected with a heritable trait may be distantly related. Conversely, the estimated (unrestricted) *F *value for rs380390 was -0.07 in control individuals.

So far, the expressions for the genotype frequencies in Table 1 simply amount to a specific formulation of the two df's associated with the three frequencies. Several other such parametrizations have previously been proposed [12]. The current transformation (1) of genotype frequencies does not by itself yield any new insights. However, in the next section we impose restrictions on the range of parameter values, which will result in a new test.

### Statistical test

We want to test the null hypothesis *H*_0 _of no association versus the alternative hypothesis *H*_1 _of association. Under *H*_0_, allele frequencies and *F *values are the same in case and control individuals while under *H*_1_, allele frequencies may be different between cases and controls and so may be *F *values. Thus, this parametrization allows testing for allele frequency differences but does so by working with genotype frequencies. To make our test as powerful as possible, we restrict estimates of inbreeding coefficients to non-negative values as these are expected to occur preferentially under *H*_1_. As outlined in detail in the Methods section, we formulate this test as a likelihood ratio (LR) test, the *FP *test. The log likelihood for case individuals is given by $\log[L_{a}(p_{a},F_{a})]=\sum_{i=1}^{3}n_{i}\log(q_{i})$ where *n*_1_, *n*_2_, and *n*_3 _are the respective numbers of case individuals with genotypes *AA*, *AB*, and *BB*, the *q*_i _are functions of *F *and *p *(Table 1), *p*_*a *_and *F*_*a *_are the parameter values in case individuals. For control individuals, the log likelihood log [*L*_*b*_(*p*_*b*_, *F*_*b*_)] is obtained in an analogous manner. The test statistic is T = 2{log [*L*_*a*_(*p*_*a*_, *F*_*a*_)] + log [*L*_*b*_(*p*_*b*_, *F*_*b*_)] - log [*L*_*c*_(*p*_*c*_, *F*_*c*_)]}, where the subscript *c *refers to the combined data (case and control individual), that is, common parameter values. For unrestricted parameter values, the test statistic *T *has an asymptotic chi-square distribution under *H*_0_. However, because of the conditions imposed (*F *≥ 0), *T *does not follow a chi-square distribution. Therefore, as outlined in the Methods section, we compute associated significance levels numerically by computer-based permutation testing. Another reason for applying permutation tests is as follows. This single test may replace the conventional two association tests (allele and genotype test) in genome-wide association studies, where 100,000s of markers are used. Test results for these markers are not independent, which is optimally taken into account in permutation tests but would be difficult to capture analytically.

### Power calculations

To evaluate the performance of our new test with existing tests, we carry out power calculations under a recessive and a dominant model of disease inheritance, where we assume a functional SNP fully associated with the disease variant. Model parameters (penetrances and disease allele frequencies) are calibrated to predict a trait prevalence of 5% for each model. The proportion of affected individuals in the population whose disease is due to the given gene is fixed at 10%. The "strength" of a model is measured by the penetrance ratio, *γ*, where *γ *= 1 corresponds to the null hypothesis.

We compare three tests, our *FP *test, the chi-square genotype test based on a 2 × 3 table of SNP genotypes versus case and control individuals, and the chi-square allele test based on a 2 × 2 table of SNP alleles. Power calculations are carried out for a type I error (rate of false positive results) of 0.05 and assumed numbers of observations of 100 case and 100 control individuals. In practice, most researchers carry out both, the allele and the genotype test, and emphasize whichever result has a smaller *p*-value without correcting for the effects of multiple testing inherent in this procedure. Thus, we capture the statistical properties of this practice by formulating a test statistic, *MaxGA*, which is the smaller of the two *p*-values associated with the genotype and allele tests. Under our model assumptions, if a result is declared significant whenever either the allele test or genotype test is significant, this practice has a type I error of 0.076 for recessive traits and 0.059 for dominant traits. In our power calculations, of course, a type I error of 0.05 is imposed for the *MaxGA *test statistic, that is, a critical limit is chosen such that the "power" of the *MaxGA *statistic is equal to 0.05 under *H*_0_.

**Figure 1:**
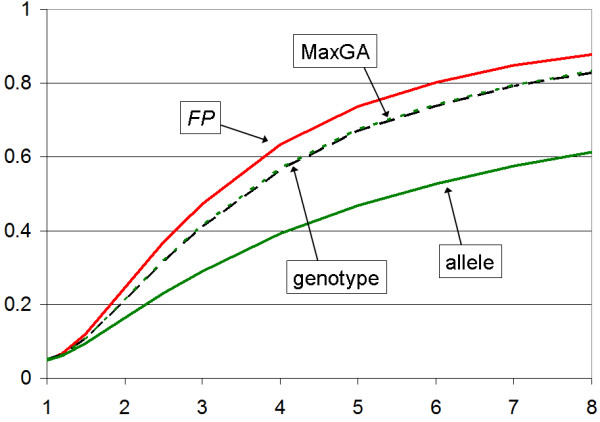
**Power for recessive disease models**. Power (y-axis) as a function of the penetrance ratio, *γ*, (x-axis), for recessive disease model. The FP test is most powerful while the MaxGA test (- - -) is slightly more powerful than the genotype test (--- --- ---).

**Figure 2:**
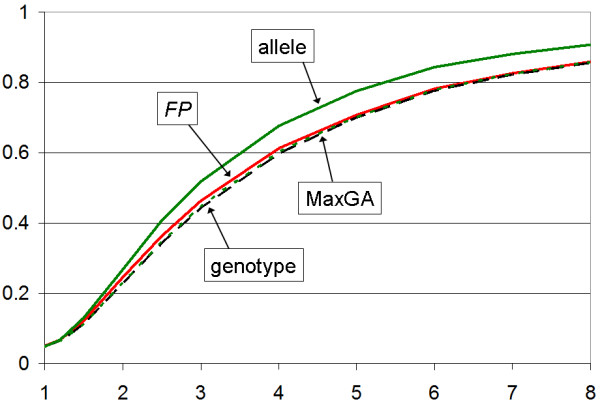
**Power for dominant disease models**. Power (y-axis) as a function of the penetrance ratio, *γ*, (x-axis), for recessive disease model. The allele test, if carried out by itself, is most powerful but, if used in conjunction with the genotype test (MaxGA), is somewhat less powerful than the *FP *test. All three tests (genotype, *FP*, MaxGA) have essentially the same power.

Figure 1 shows power curves for recessive disease models. Clearly, our *FP *test has better power than any of the other association tests. For dominant traits (Figure 2), if researchers carried out only the allele test, that test has higher power than the *FP *and genotype tests. As both the allele and genotype tests are usually carried out at the same time, the proper comparison is between the *MaxGA *statistic and our *FP *test, where the latter exhibits a slight advantage over the former. Thus, it is fair to say that the *FP *test is more powerful than conventional test statistics (allele and genotype tests) under our recessive and dominant inheritance models. We have implemented it in a computer program, *FPtest*, which is freely available on our website [13].

Under both dominant and recessive models, the *MaxGA *statistic has essentially the same power as the genotype test. Thus, any superiority of the allele test, if carried out at the same time as the genotype test, is wiped out by the multiple testing correction. As the usual practice is to do both, an allele and a genotype test, the potentially higher power of the allele test is fallacious as it is entirely purchased by an increased rate of false positive results.

We also considered Risch's genotype relative risk model [14], in which the penetrances for the three disease genotypes are given by *f*, *γf*, and *γ*^2^*f *so that this model may be viewed as being intermediate between dominant and recessive inheritance. For our assumptions on disease inheritance, this model always predicts unrestricted parameter values of *F *= 0 in case and slightly negative *F *values in control individuals, where the latter would be estimated to be zero under our restrictions. Thus, the *FP *test is expected to essentially default to the genotype test under Risch's penetrance structure but this is not further pursued here.

### Application to published data

We applied our *FP *test to published data on age-related macular degeneration (AMD) [11,15] and Parkinson disease (PD) [16,17]. In these genome-wide studies, the number of SNPs ranged from 100,000 (AMD) to 393,000 (PD). The empirical (experiment-wise) significance level associated with each association test was estimated in 5,000 randomization samples (labels "case" and "control" randomly permuted) as the proportion of such samples exhibiting a test statistic at least as large as the observed test statistic for any one of the SNPs. Table 2 shows the results of the genotype and *FP *tests in these published data. For completeness, results for the allele test are also shown. Significance levels in a given row of Table 2 were computed on the basis of the same set of randomization samples but different samples were used for different rows. For these calculations, we used the permutation procedure [18] implemented in our *sumstat *program [19], which employs the most recently published 64 bit random number generator [20].

**Table 2:** Test results for observed data

Data	SNP	*p*_genotype_	*p*_FP_	*p*_allele_	*F*_case_	*F*_control_
AMD	rs380390	0.0380	0.0090	0.0056	0.215	-0.073
	rs10272438	1.0000	0.9068	0.0194	0.733	0.611

AMD HK	rs10490924	0.0002	0.0002	0.0002	0.243	-0.062
	rs10504152	0.0058	0.1286	0.2222	0.132	-0.271
	rs584244	0.1824	0.0996	0.1010	-0.011	-0.149

PD	rs9952724	0.0004	0.0002	1.0000	0.788	0.022
	rs850084	0.0022	0.0002	0.9932	0.828	0.243
	rs10963676	0.0058	0.0004	0.0072	0.817	0.086
	rs4746675	0.0062	0.0004	1.0000	0.839	0.048
	rs557074	0.0068	0.0012	1.0000	0.736	0.029
	rs1504212	0.0088	0.0014	1.0000	0.494	-0.023
	rs12364577	0.0174	0.0020	1.0000	0.519	0.014
	rs1468375	0.0240	0.0042	0.0002	0.452	-0.040

Results of the genotype, *FP*, and allele tests applied to three published datasets: AMD in Caucasians [11], AMD HK in data from Hong Kong [15], and PD [16]. Estimated *F *values based on observed numbers of genotypes in case and control individuals are unrestricted, that is, may be negative.

For each of the three studies in Table 3, all SNPs are listed that achieved an experiment-wise significance level of 0.05 or less in either one of the three association tests. Of the 13 resulting SNPs, 11 show a smaller *p*-value for the *FP *test than the genotype test and one SNP shows the same *p*-value. These results clearly demonstrate the usefulness of our new association test. As expected, observed unrestricted *F *values are larger in case than control individuals and in the latter are often negative.

## Discussion

As mentioned in the introduction, researchers often look for genomic regions of increased homozygosity or autozygosity by sliding a window of fixed length across the genome. Our test offers an elegant alternative to such windows of fixed and arbitrary lengths. We propose to work with scan statistics as previously developed [21]. This method also employs a window of a fixed length (fixed number of SNPs) and determines the maximum of the sum of test statistics for all such windows in the genome, which is the scan statistic of the given length. What sets this approach apart from ad hoc approaches is that it applies different window sizes from 1 up to a specified maximum length and estimates optimal window length by maximum likelihood. An updated version of our *scanstat *program is available that incorporates the *FP *test statistic [22]. This implementation allows users to determine the most significant stretch of continguous markers with high values of the *FP *statistic, which we interpret as a genomic region of high IBD.

It is interesting to note unrestricted values of *F *predicted by our disease models. For example, for *γ *= 5, the recessive model predicts 0.316 in cases and -0.025 in controls. This explains why our *FP *test has higher power than conventional tests for recessive traits: Inbreeding coefficients tend to be strongly positive in cases and only slightly negative in controls (this implies strong deviations from HWE in cases). Our parameter restrictions disallow negative *F *values, which reduces "noise" in the determination of significance levels. On the other hand, the dominant model predicts unrestricted *F *values of -0.041 in cases and 0.002 in controls. These values are only slightly different from 0 and will become non-negative in the *FP *test, that is, the *FP *essentially defaults to the genotype test with only a slight advantage over it.

The null distribution of the *FP *test statistic (under *F *≥ 0) is not known and would be difficult to obtain, particularly for large numbers of markers whose test results are non-independent.

## Conclusion

At least for the recessive and dominant models considered here, our *FP *test is more powerful than allele and genotype tests. Thus, it represents an attractive alternative to these conventional tests. A potential disadvantage of the *FP *test might be that it requires permutation testing for an appropriate determination of *p*-values. However, permutation testing is one of the best approaches to correct for multiple testing in genome-wide association studies and is often carried out anyway, so the *FP *test does not represent an additional burden.

## Methods

**Figure 3:**
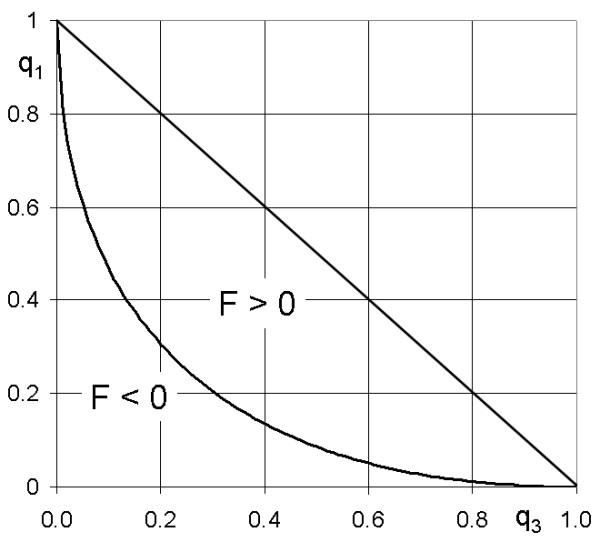
**Parameter space for SNP genotype frequencies**. Parameters *q*_1 _and *q*_3 _are frequencies for genotypes *AA *and *BB*, respectively; *F *= probability of autozygosity, inbreeding coefficient.

The restriction of *F *and *p *to non-negative values reduces the parameter space of genotype frequencies, *q*_1 _and *q*_3_. Simple algebraic manipulation (not shown here) of equations (1) demonstrates that 0 ≤ *p *≤ 1 is always satisfied and so is *F *≤ 1. However, *F *≥ 0 is only satisfied for 2*p*(1 - *p*) ≥ *q*_2_. Figure 3 shows the parameter space for the three SNP genotypes. Because of *q*_1 _+ *q*_3 _≤ 1, the unrestricted parameter space corresponds to the lower triangle in Figure 1. The requirement that F ≥ 0 translates into the inequality,

$$q_{1}\geq 1+q_{3}-\sqrt{{\left(1+q_{3}\right)}^{2}+q_{3}(2-q_{3})-1}.$$

The resulting restricted parameter space corresponds to the area marked "F > 0" in Figure 3, that is, the area between the convex solid line and the diagonal. The restricted parameter space is only 2/3 of the surface of the unrestricted parameter space.

For power calculations, we assume disease models with two alleles and three genotypes, *dd*, *Dd*, and *DD*. The respective penetrances are *f*_1_, *f*_2_, and *f*_3_, where we set *f*_2 _= *f*_1 _for recessive models and *f*_2 _= *f*_3 _for dominant models. The "strength" of a model is measured by the penetrance ratio, *γ *= *f*_3_/*f*_1_, which is very approximately equal to the odds ratio. Thus, we have three genetic parameters, *p*, *f*_1_, and *γ*, which predict trait prevalence as

*K *= *γf*_1_*p*^2 ^+ *f*_1_(1 - *p*^2^) for recessive traits and as

*K *= *q*^2^*f*_1 _+ (1-*q*^2^)*γf*_1_, *q *= 1 - *p*, for dominant traits.

Also, the proportion of genetic cases among all affected individuals is

*Q *= *γp*^2^/(*γp*^2 ^+ 1 - *p*^2^) for recessive traits and

*Q *= (1 -*q*^2^)*γ*/[(1 - *q*^2^)*γ *+ *q*^2^] for dominant traits.

Fixing *K *= 0.05 and *Q *= 0.10 leaves one free parameter, which we vary to generate power curves. For a fixed set of genetic parameter values, Bayesian calculations yield conditional genotype frequencies in case and control individuals, from which random samples (replicates) are drawn.

For each of dominant and recessive models, with a value of the penetrance ratio, *γ *= 1, critical limits for test statistics are chosen so as to make the type I error for each of them equal to 0.05. That is, the critical limits are chosen such that the proportion of randomization samples exceeding this limit is equal to 0.05 (in other words, we are using the 95^th ^percentile of the computer-generated null distribution of the test statistic as the critical limit). Then power is determined for penetrance ratios ranging from 1 through 8. All power calculations were carried out based on 5,000 replicates.

## Authors' contributions

QZ participated in study design and programming, SW formulated the parametrization of SNP genotypes, and JO participated in study design and wrote the manuscript.
